# A new surgical technique: transvesical resection of prostate - case series

**DOI:** 10.1590/S1677-5538.IBJU.2018.0113

**Published:** 2018

**Authors:** Hakan Türk, Sitki Ün, Erkan Arslan

**Affiliations:** 1 Department of Urology, Evliya Celebi Training and Research Hospital, Kutahya, Turkey; 2 Department of Urology, Sivas State of Hospital, Sivas, Turkey; 3 Department of Urology, Harran University Medical School, Sanliurfa, Turkey

**Keywords:** Transurethral Resection of Prostate, Urethra, Urinary Bladder Neck Obstruction

## Abstract

**Objective:**

To protect the urethra from instrumentation related urethra injures and stricture, we developed a new surgical technique which can be defined as transvesical resection of prostate without using urethra.

**Materials and Methods:**

Our study included 12 consecutive bladder outlet obstruction patients treated with transvesical prostate resection in our clinic between March 2016 and May 2016. Detailed anamnesis, results of physical examination, digital rectal examination, routine lab tests, international prostate symptoms score, transrectal ultrasound, measurement of prostate-specific antigen levels and uroflowmetry was performed in all patients prior to surgery.

**Results:**

Hospitalization period following surgery was 1 day. Foley catheter and suprapubic cystostomy catheters were removed in a median period of 3.6 days and 1 day. Median mass of resected adenomas was measured as 21.8 gr. Median maximum flow rate was measured as 6mL/s. Median postvoid residual urine volume was 70.6 cc and median international prostate symptoms score and quality of life scores were 9 and 1.4, respectively.

**Conclusion:**

In this study, we would like to show the possible practicality of transvesical resection of prostate technique in this patient group. However, we think that this technique is very useful in special patient groups such as patients with bladder stones, priapism and penile prosthesis.

## INTRODUCTION

**Figure 1:**
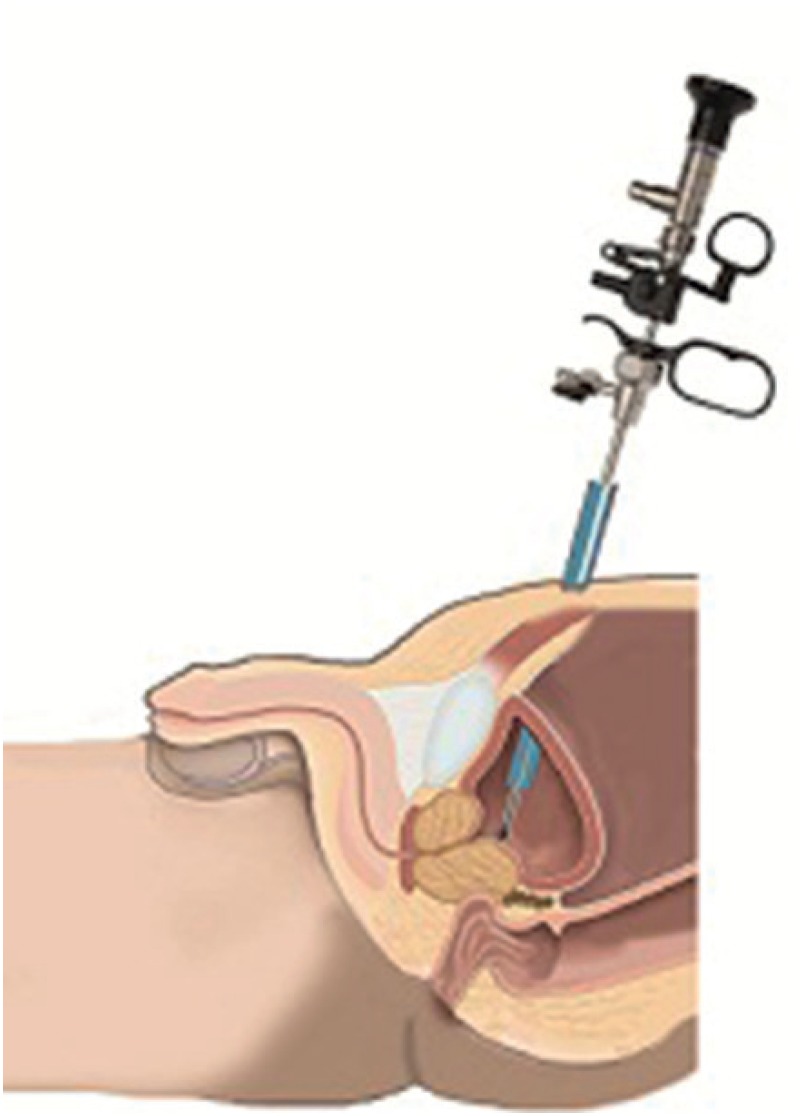
Percutaneous puncture of the bladder and placement of the renal sheath and resectoscope.

Bladder outlet obstruction (BOO) is a common condition seen in males over 40 and its incidence rate shows an increase with age. The prevalence of BOO is measured at around 8% in males at age 40, but it increases to almost 90% in males over 90 (1). Medical treatment for relieving BOO - related lower urinary tract symptoms is the first line of treatment in those cases. However, surgical intervention is recommended for patients who did not benefit from medical treatment or for patients with BOO - related complications such as recurring urinary tract infections, acute retention, hematuria, and bladder stones (2). When surgical intervention techniques are considered, a transurethral prostate incision is used in patients with prostate volumes < 30 cc, and a transurethral prostate resection (TURP) is chosen for patients with prostate volumes between 30 and 80 cc. Finally, an open prostatectomy procedure is recommended for patients with prostate volumes > 80 cc (3). Other options include Holmium - laser enucleation of the prostate and laparoscopic removal of adenoma tissues from the prostate (4-6). However, all the surgical techniques mentioned above have different early - and late - term complication risks. Different studies have reported post - TURP urethral stricture as 2.2 – 9.8% and bladder neck stricture between 0.3% and 9.2% (7-9). The main reason for those complications is thought to be the scar formation caused by mucosal lacerations during the use of the urethra for surgery (10). However, in daily practice, difficulties are experienced in special situations where transurethral prostatectomy is a challenge, such as with patients with penile prosthesis, patients with bladder stones, patients who develop priapism during surgery, and patients who cannot be positioned due to hip issues. In this study, a new technique (transvesical prostate resection) for prostate resection is defined and its outcomes and practicality are assessed (Figure-1).

## MATERIALS AND METHODS

This study was approved by the Ethics Committee of the University of Harran (17.09.2017, Meeting: 09 / Decision: 1), and the written, informed consent of all patients was obtained. Our study included the observation of 12 consecutive BOO patients treated with transvesical prostate resection (TVRP) in our clinic between March 2016 and May 2016. All surgeries were performed by the same surgeon. Patient inclusion criteria included a prostate volume of at least 30 g, a recurring acute urinary retention or maximum flow rate (Qmax) < 10 mL / s, and an international prostate symptom score (IPSS) of at least 12. Detailed patient history, results of physical examination, digital rectal examinations, routine lab tests, IPSSs, measurements of prostate - specific antigen (PSA) levels, and uroflowmetry results were recorded in all patients prior to surgery. For comparison with pre - operative results, IPSSs and uroflowmetry were repeated 1month, 3 months, and 1 year following surgery. In patients with PSA values of 4 or more or with other risk factors (such as nodules observed during a digital rectal examination), a prostate biopsy was also performed prior to surgery to eliminate the possibility of cancer. A histopathology examination of all samples confirmed benign prostatic hyperplasia (BPH) in all cases.

Statistical analysis was performed using IBM's SPSS (Statistics for Windows, version 22.0., Armonk, NY, USA) program. Cases were divided into two groups and a definite data analysis (mean, median, range and percentages) was performed. Two sample T - tests were used for intergroup comparisons. Statistical significance level was set as p < 0.05.

### Equipment Used in Surgery

All operations were done under either general or spinal anesthesia, and a bipolar resectoscope was used. A bipolar TVRP (Olympus 24 -channel rotating continuous flow type from Tokyo, Japan) was used in a 200 W setting for resection and a 100 W setting for coagulation. Continuous irrigation was done during the TVRP, and 0.9% saline solution bags were hung at the lowest height allowable for proper fluid flow (max 60 cm).

#### Surgical Technique

All interventions were done in the lithotomy position (except for the first patient due to a hip prosthesis) under general or spinal anesthesia. No issues were detected when surgery was performed in the supine position. However, most of the patients were operated on in the lithotomy position for quick intervention in case of any complications during the first cases where the technique was used.

Just prior to surgery, a cystoscopy was performed using a 17 FR scope to rule out possible comorbidities such as bladder cancer or urethral stricture, and to assess the resection percentage of the prostate. The procedure started after completely filling the bladder with a saline solution to ease suprapubic access. To ensure continuous intravesical guidance during the percutaneous approach, the front wall of the bladder was examined using an endoscope. Bladder entry was done by inserting an access needle from the suprapubic midline, 4 cm above the symphysis pubis, through the skin.

**Figure 2:**
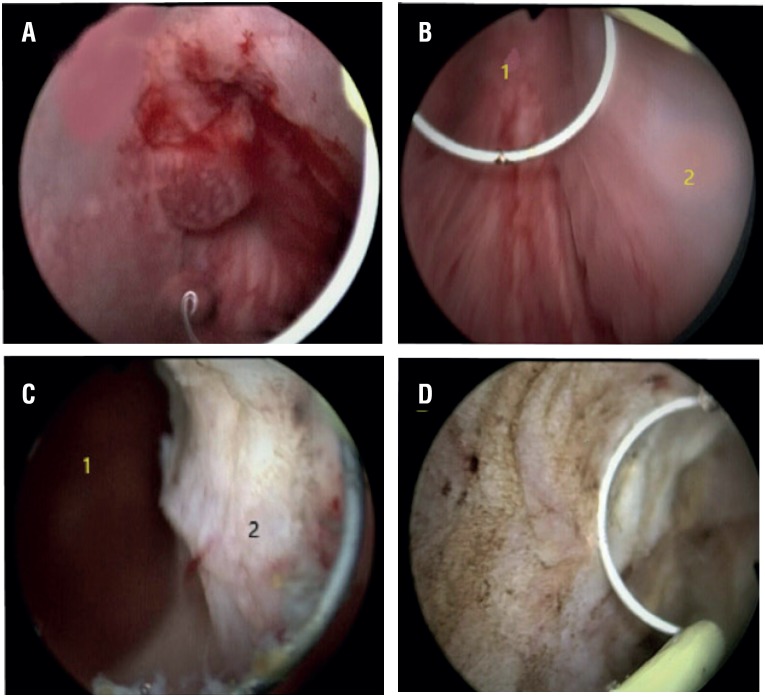
A) Retrograde view of bladder neck and intravesical prostatic protrusion, B) 1- Verrumoontanum, 2- Right lateral lobe, c) 1- verrumoontanum, 2- Resected right lateral lobe, D) Resected left lateral lobe.

After proper insertion of the guide wire, the canal was dilated using a 12 Fr Amplatz dilator, and the canal was formed by using a 28 – 30 Fr Amplatz dilator (one - shot technique). A renal sheath was inserted into the formed canal. Then, using a 24 FR resectoscope shaft, the bladder was entered through the renal sheath. The bladder neck and the prostate were identified. The prostate resection began from the middle lobe (if present). It continued on to the ventral sides (between the directions of 11 and 1 o'clock), then to both lateral lobes, and finished with the apex (Figure-2).

**Figure 3:**
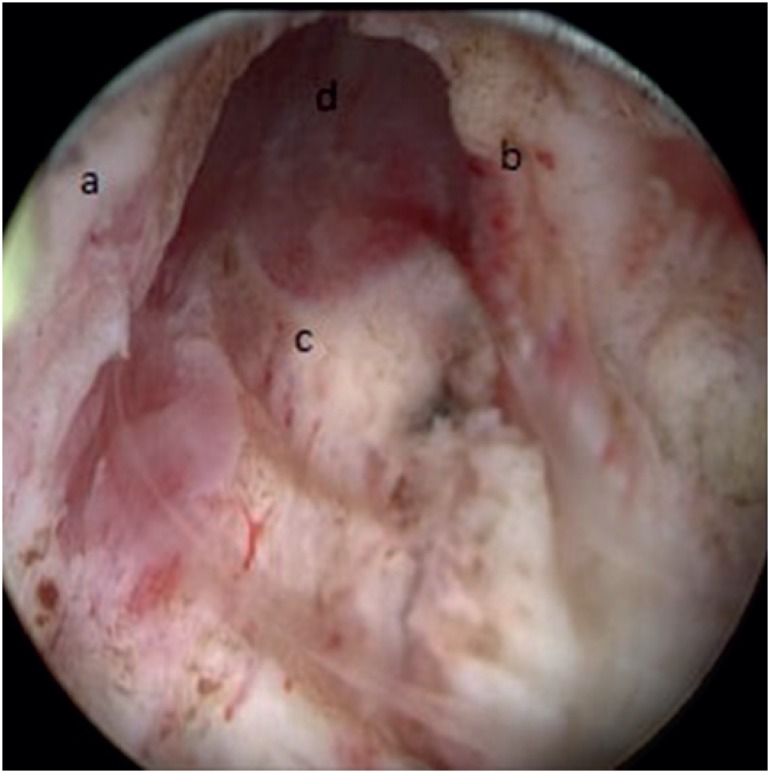
Retrograde view of resected prostate. a) Resected right lateral lobe, b) Resected left lateral lobe, c) Resected median lobe, d) Verrumoontanum.

In order to get a better view of the apex and the sphincter, a 18 Fr Foley catheter was inserted, and the tip of the Foley catheter was used as a land mark point in some cases. Lateral, anterior, and apical prostate tissues were resected to the prostate capsule (Figure-3). This method allowed an almost unnoticeable angle between the prostate and the urethra, which did not affect the surgical intervention at all. Second generation intravenous cephalosporin was used for prophylaxis in all patients. An 18 Fr two - way urethral Foley catheter was placed at the end of surgery for drainage. Following the insertion of a 16 Fr nelaton catheter through the renal sheath, it was attached to the skin using 3 – 0 vicryl.

In the cases where irrigation was necessary, irrigation fluid was introduced via a suprapubic nelaton catheter. Urethral catheters were removed at the 3^rd^ – 5^th^ day of surgery after urine output became clear in color, except for patients who developed complications such as hematuria or clot retention. The suprapubic catheter was removed 1day after surgery and patients were discharged. In all patients, complete blood count and serum electrolytes were measured after the surgery. Signs and symptoms of possible transurethral resection syndrome were also clinically reviewed. In addition, postoperative and perioperative complications, operation period, resected prostate tissue mass, catheter removal time, and hospitalization period were all recorded on file. Post - op assessments were done in the first month, third month and first year following surgery. During the follow-up period, uroflowmetry, postvoid residual urine volume measurements via ultrasonography, IPSSs, and quality of life (QoL) scores were measured for post - op assessment.

## RESULTS

All TVRP surgeries were successfully completed without any complications that might have required the surgery to be changed to a standard TURP or an open prostatectomy. The hospitalization period following surgery was 1 day in all patients. The Foley catheter was removed within 3 to 5 days after surgery, and the suprapubic catheter was removed the next day after surgery. The preoperative median maximum flowrate (Qmax) was 5.5 (4 – 10) mL / s. The median preoperative postvoid residual urine volume was 77.5 (20 – 100) cc and the median IPSS and QoL scores were calculated as 23.5 and 5, respectively. A month after the operation, the median Qmax increased to 17.5 (12 – 28) cc / s, and the median postvoid residual urine volume was 52.5 (30 – 75) cc. The median IPSSs and QoL scores were found to be 9.5 and 1, respectively (Table-1). The median resected adenoma mass was 22.5 (18 – 45) gr. No major complications related to surgery or anesthesia were seen. None of the patients developed TUR syndrome. Only 1 patient developed erysipelas at the cystostomy entry point and was treated with first generation cephalosporin. There were 2 patients who developed stress incontinence within the first month of surgery and were relieved after a month of Kegel exercises. Only 1 patient developed epididymo - orchitis on the seventh day of surgery and was treated with second generation cephalosporin. A new catheter was placed in a patient who used anticoagulants for coronary artery disease after seeing macroscopic hematuria 5 days after the Foley catheter was removed. Hematuria was cleared with irrigation and the catheter was removed 3 days later. None of the patients developed urethral stricture in their mean 1 - year follow-up period.

**Table 1:** Comparison of preoperative and postoperative data.

Preoperative					Postoperative				
**Case**	**IPSS**	**QoL**	**Q MAX (mL/sn)**	**PVR (mL)**	**IPSS**	**QoL**	**QMAX (mL/sn)**	**PVR (mL)**	**Complications**
**1**	AUR	AUR	AUR	AUR	12	2	12	50	
**2**	AUR	AUR	AUR	AUR	11	3	15	80	
**3**	22	5	7	80	10	2	17	60	Hematuria
**4**	25	5	4	100	5	1	28	75	Epididymo-orchiditis
**5**	18	5	10	20	7	2	18	30	Erysipelas
**6**	25	5	5	50	10	1	19	30	Stress Incontinence
**7**	22	5	4	100	8	1	18	60	
**8**		Bladder Stone	Bladder Stone	Bladder Stone	10	2	18	45	
**9**	AUR	AUR	AUR	AUR	13	1	17	40	Stress Incontinence
**10**	20	5	6	60	8	1	16	55	
**11**	28	6	5	75	6	0	20	30	
**12**	30	5	7	80	9	1	17	60	
**Median**	23.5	5	5.5	77.5	9.5	1	17.5	52.5	

**IPSS =** International Prostate Symptom Score; **QoL =** Quality of life; **Qmax =** Maximal urine flow rate; **PVR =** Post-Void Residual urine; **AUR =** Acute Urinary Retention

Except for 4 patients with urinary retention and bladder stones prior to surgery, IPSSs and QoL scores increased significantly with the surgery (Table-2). In addition, except for the 4 patients mentioned above, there were significant improvements in maximum flow rates and postvoid residual urine volumes following surgery (Tables 1 and 2).

**Table 2:** Preoperative and postoperative data expressed as median.

	Preoperative	Postoperative	P Value
IPSS	23.5	9.5	<0.001
QoL	5	1	<0.001
QMax (mL/s)	5.5	17.5	<0.001
PVR (mL)	77.5	52.5	<0.001

**IPSS =** International Prostate Symptom Score; **QoL =** Quality of life; **Qmax** = Maximal urine flow rate; **PVR =** Post-Void Residual urine

## DISCUSSION

The main factors that affect surgical treatments are the patient's age, health status, expectations of surgery results, and prostate volume. TURPs, open prostatectomies, transurethral prostate incisions, and finally, transurethral prostate electro - vaporizations were defined as conventional surgery techniques by the European Association of Urology (11). Transurethral resection continues to be the golden standard for the surgical management of bladder outlet obstructions throughout the years. TURP can improve lower urinary tract symptoms up to 70%, but it comes with a morbidity risk of 20%. Urethral stricture is one of the most important postoperative complications of a TURP. Different series reported urethral stricture incidence rates between 2.2% and 9.8% (8-12).

Urethral stricture continues to be a challenge for urology for its repetitive nature, the need for patient care, treatment difficulties, and follow-up problems. The main causes thought to play a role in urethral stricture development are large prostate volumes and related longer operation periods, the size of the catheter used, infected urine, usage of a thick resectoscope with high energy, unnecessary insertions and removals of the resectoscope, insufficient lubrication of the resectoscope (which causes friction in the bulbous urethra from the penoscrotal angle), and energy outbursts that develop on the resectoscope shaft, damaging the urethra mucosa (7, 13, 14). About 60% of strictures redevelop within a year after the initial treatment, which requires complex interventions such as urethroplasty and dilatation. This complication risk is severe enough to worry about urethral stricture developing following TURP.

In our technique, the urethra is not used, and the prostate is removed through the bladder, similar to open prostatectomies. For this reason, we believe that it has an advantage over TURP for urethral stricture development. However, prospective studies with longer patient series are necessary to make a clear comparison between our technique and other those methods and validate our results. The urethral stricture occurrence rate in open prostatectomy is reported as 1.9% (7, 15). The most important reason for this is not using the urethra during surgery. In a TVRP, the urethra is not used, except for very short periods of time while using thin instruments for checking the bladder and urethra. The fact that none of the patients developed a urethral stricture during the mean follow-up period of 1year supports our thesis that the risk of urethral stricture can be reduced by using our method.

Penile tumescence and priapism can be seen due to anesthetic agents used in spinal or general anesthesia, or due to an imbalance between sympathetic and parasympathetic nerve systems (16). This situation can cause interruptions in transurethral approaches or a complete cancellation of surgery (16). The main reason for this is the hemorrhage risk and urethral trauma caused by the transurethral operation. Similarly, transurethral passing of the resectoscope can be difficult in patients with penile prosthesis implants (17). In such situations, although different approaches such as transperineal urethral resections are possible, those approaches increase the complication risks during the post - operative period. Also, in patients that cannot be placed in a lithotomy position due to having a hip prosthesis, transurethral techniques are almost impossible to perform. For those patients, open prostatectomy is preferred. However, TVRP can also be performed in the supine position, which is advantageous for this patient group. TVRP can be used in these situations to provide a barrier for such complications. TVRP seems more advantageous over TURP in this sense. Except for one patient, all patients included in this study were operated on in the lithotomy position. The main rationale behind this was to enable faster intervention for possible complications that could arise during the first few uses of this new technique. However, as our experience increases, we will start operating on patients in the supine position as well.

Bladder stones, caused by a bladder blockage related to BOO, can be seen in 5% of all urinary tract stones (18). This might cause an additional cystolithotomy operation prior to prostate surgery. This situation complicates the surgery in patients with multiple or large bladder stones. Open, transurethral and percutaneous cystolithotripsy are the most used methods for bladder stones (19, 20). Recent publications report a preference of percutaneous over transurethral cystolithotripsy for bladder stones due to urinary stricture risks and shorter operation times (19-22). In this technique we developed, both surgeries can be percutaneously performed from the same entry point. We used the TVRP method for the first time on a patient who had bladder stones and could not be placed in a lithotomy position due to a hip prosthesis. After successfully treating this patient, we used the same method, as planned, for our other TURP patients. In patients with BOO - related bladder stone formations, an intervention from a single - entry point to both the bladder stone and the prostate can be beneficial. However, prospective comparative studies with larger patient series are necessary to make a clear statement about effectiveness of TVRP.

Since this technique has been developed and used only by us, we cannot compare our results to any other previous studies. However, studies assessing the efficacy of TURP reported Qmax improvement by + 162%, significant decrease in IPSS by – 70%, significant decrease in QoL scores by – 69% and decrease in post - voiding residual urine volume by – 77% (23, 24). In our technique, we observed an increase in Qmax by + 298%, a decrease in IPSS by – 62.1%, decrease in QoL by −69% and decrease in post - voiding residual urine volume by – 27.5%. When our results were compared with those results in the literature, related results show the postoperative efficacy of TVRP. However, the low number of patients in the assessment of early and late - term post - operative complications is one of the limitations of our study.

**Figure 4:**
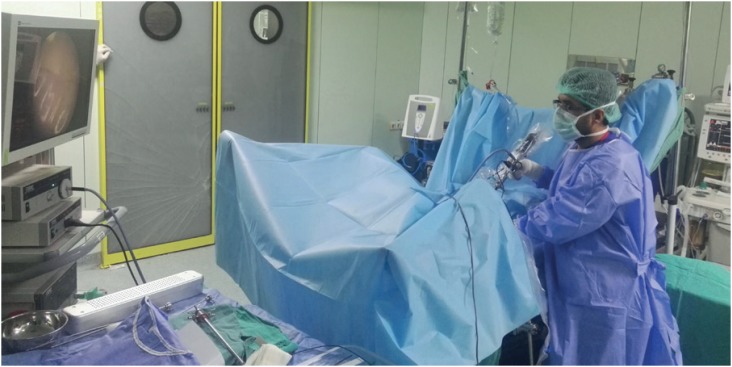
Position of the surgeon at the left side of patient.

The main limitations in this study where we explain a newly developed technique include a low number of patients, and insufficient follow-up periods to assess complications such as urethral strictures and bladder neck strictures. In addition, the inability to use this technique during the surgery on BOO patients with coexisting bladder tumors can also be considered a limitation. The technique can be challenging in cases with prostate volumes over 80 cc as it is a relatively new method. Moreover, it might be impossible to place the resectoscope in the desired angle in morbidly obese patients due to the fat tissue on the anterior abdominal wall. Finally, in the transurethral method, surgeons are positioned exactly at the patient's midline (lithotomy position), whereas in our technique, surgeons are positioned on the left side of the patients, which can be considered a difficulty (Figure-4).

Claiming that TVRP is as effective as TURP is a relatively bold statement that is based on a single study. Prospective comparison studies with larger patient series are necessary for the clarification of this subject.

## CONCLUSIONS

In this study, we would like to show the possible practicality of the TVRP technique in patients with BOO. We think that this technique is very useful and has potential for daily practice, as it has a low risk of urethral stricture development, does not employ the urethra and is utilizable in special patient groups (such as patients with bladder stones, priapism and penile prosthesis). For a clear assessment of urethral stricture developments in such cases, larger series with longer patient follow-up periods are required.
